# Prognostic and Clinicopathological Value of Programmed Cell Death Ligand1 Expression in Patients With Small Cell Lung Cancer: A Meta-Analysis

**DOI:** 10.3389/fonc.2020.01079

**Published:** 2020-06-25

**Authors:** Huarong Cai, Haimei Zhang, Yuequan Jiang

**Affiliations:** ^1^Department of Thoracic Surgery, Chongqing University Cancer Hospital, Chongqing Cancer Institute, Chongqing Cancer Hospital, Chongqing, China; ^2^Department of Urology, Chongqing University Cancer Hospital, Chongqing Cancer Institute, Chongqing Cancer Hospital, Chongqing, China

**Keywords:** PD-L1, SCLC, meta-analysis, immunotherapy, risk factor

## Abstract

**Background:** Programmed death-ligand 1 (PD-L1) is an immune checkpoint molecule expressed by cancer cells. Previous studies have demonstrated the prognostic role of PD-L1 expression in patients with small cell lung cancer (SCLC), where the results were inconsistent. Therefore, we conducted a meta-analysis to identify the prognostic impact of PD-L1 on SCLC.

**Methods:** We searched the PubMed, Embase, ISI Web of Science, and Cochrane Library databases for articles published before and on March 2nd, 2020. Data of PD-L1 expression in tumor cells detected using immunohistochemistry methods were extracted for analysis. Pooled hazard ratios (HRs) with confidence intervals (CIs) and odds ratios (ORs) with 95% CIs were calculated to assess the correlations among PD-L1, overall survival (OS), and clinicopathological factors.

**Results:** Nine studies of 921 patients published between 2015 and 2019 were included in this meta-analysis. The pooled data (HR = 0.91, 95% CI = 0.46–1.80, *p* = 0.787) indicated that PD-L1 expression is not a significant predictor of poor OS. Moreover, the results also revealed that PD-L1 expression is not significantly associated with gender (OR = 1.12, 95% CI = 0.73–1.74, *p* = 0.601), age (OR = 1.15, 95% CI = 0.58–2.30, *p* = 0.683), pN stage (OR = 0.65, 95% CI = 0.24–1.72, *p* = 0.381), pT stage (OR = 1.16, 95% CI = 0.26–5.23, *p* = 0.847), serum lactate dehydrogenase level (OR = 1.06, 95% CI = 0.13–8.43, *p* = 0.958), or performance status (OR = 0.69, 95% CI = 0.24–1.95, *p* = 0.479). No significant publication bias was detected in this meta-analysis.

**Conclusions:** This meta-analysis suggests that PD-L1 expression is not a significant prognostic factor of poor survival in SCLC. Because of significant variations, high-quality studies are needed to validate our results.

## Introduction

Lung cancer remains the leading cause of cancer-related death in both men and women worldwide (1). Small cell lung cancer (SCLC) accounts for ~13% of all lung cancer cases (2) and has aggressive biological behaviors. SCLC is characterized as being sensitive to chemotherapy and radiotherapy, and emerging therapies for SCLC include immunotherapy and targeted therapy (3). Despite the progress in treatment, the prognosis of SCLC is usually poor, with a median survival of ~1 year at any stage (4). Therefore, identifying novel and effective prognostic biomarkers is crucial to formulate treatment strategies.

Immune checkpoint inhibitors (ICIs) targeting programmed cell death protein-1 (PD-1)/programmed cell death ligand-1 (PD-L1) show promising antitumor activity in patients with SCLC (5, 6). A recent randomized, controlled, phase 3 trial showed that the combination of the PD-L1 inhibitor atezolizumab and chemotherapy as the first-line treatment results in significantly longer survival than that following chemotherapy alone in patients with extensive-stage SCLC (7). Previous studies have also investigated the prognostic role of PD-L1 in patients with SCLC, which revealed inconsistent results (8–16). For example, some reports suggested that high PD-L1 expression is correlated with poor overall survival (OS) in patients with SCLC (10, 15). On the contrary, other studies demonstrated that PD-L1 overexpression is an independent prognostic factor of favorable OS in patients with SCLC (8, 13, 14). Therefore, we collected relevant articles and conducted a meta-analysis to quantitatively evaluate the prognostic and clinical value of PD-L1 in SCLC, which will also provide implications for the application of PD-1/PD-L1 inhibitors for this disease.

## Materials and Methods

### Search Strategy

This meta-analysis was performed in accordance with the Preferred Reporting Items for Systematic Reviews and Meta-Analyses (PRISMA) statement (17). We searched the electronic databases of PubMed, Embase, ISI Web of Science, and Cochrane Library for relevant reports. The last search was conducted for all articles published before and on March 2nd, 2020. The search terminologies were “programmed cell death-ligand 1, PD-L1, PDL1, CD274, B7-H1,” and “lung cancer, lung carcinoma, small cell lung cancer, small cell lung carcinoma.” All publications were written in English. The references of the retrieved relevant articles were manually checked. Ethics approval was not necessary for this meta-analysis because all data were derived from published studies.

### Inclusion and Exclusion Criteria

Qualifying studies were required to meet the following inclusion criteria: (1) the diagnosis of SCLC was confirmed by histologically and/or pathologically examination; (2) the expression of PD-L1 was detected by immunohistochemical (IHC) staining methods; (3) PD-L1 was determined in tumor cells; (4) the correlation between PD-L1 and prognosis was reported or relevant data were provided; and (5) the report was published in English. The exclusion criteria were as follows: (1) reviews, meeting abstracts, letters, or case reports; (2) duplicate publications; or (3) studies with insufficient data.

### Data Extraction and Quality Assessment

All relevant data were extracted by two independent researchers (HC and HZ), and all discrepancies were settled by discussion. The following data were extracted: first author's name, publication year, study location, study period, sample size, age, gender, treatment, cut-off value, evaluation method, study design, tumor stage, and hazard ratios (HRs) and 95% confidence intervals (CIs) for survival outcomes. If the HRs and 95% CIs were not directly reported, they were calculated according to Tierney's method (18). If PD-L1 expression was determined in tumor cells, stromal cells, and/or tumor-infiltrating lymphocytes in a study, only the data of PD-L1 expression in tumor cells detected using IHC methods were extracted for this meta-analysis. If the required data were not provided in an article, the items were labeled as “not available” (“NA”). Quality assessment of the included studies was performed using the Newcastle–Ottawa Scale (NOS) (19). The NOS mainly focuses on three parts: selection, comparability, and outcome assessment. The highest NOS score was 9 points, and when studies scored NOS ≥ 6, they were considered high-quality studies.

### Statistical Analysis

Pooled HRs with CIs were calculated to assess the correlation between PD-L1 and OS. The heterogeneity among studies was determined using a chi square-based *Q*-test (20) and *I*^2^ statistics (21). A random-effects model was used when significant heterogeneity (*I*^2^ > 50% or *P* < 0.01) was detected. A fixed-effects model was also implemented. Associations between PD-L1 and clinicopathological features were measured using the pooled odds ratio (OR) and 95% CIs. Subgroup analysis was carried out to explore the source of heterogeneity. Sensitivity analysis was conducted by omitting each study in turn to evaluate the influence of a single study on the total results. Publication bias was measured by applying Begg's and Egger's tests. All statistical analyses were performed using Stata statistical software, version 12.0 (STATA Corporation, College Station, TX, USA). Values of *p* < 0.05 were considered statistically significant.

## Results

### Study Selection

**Figure 1:**
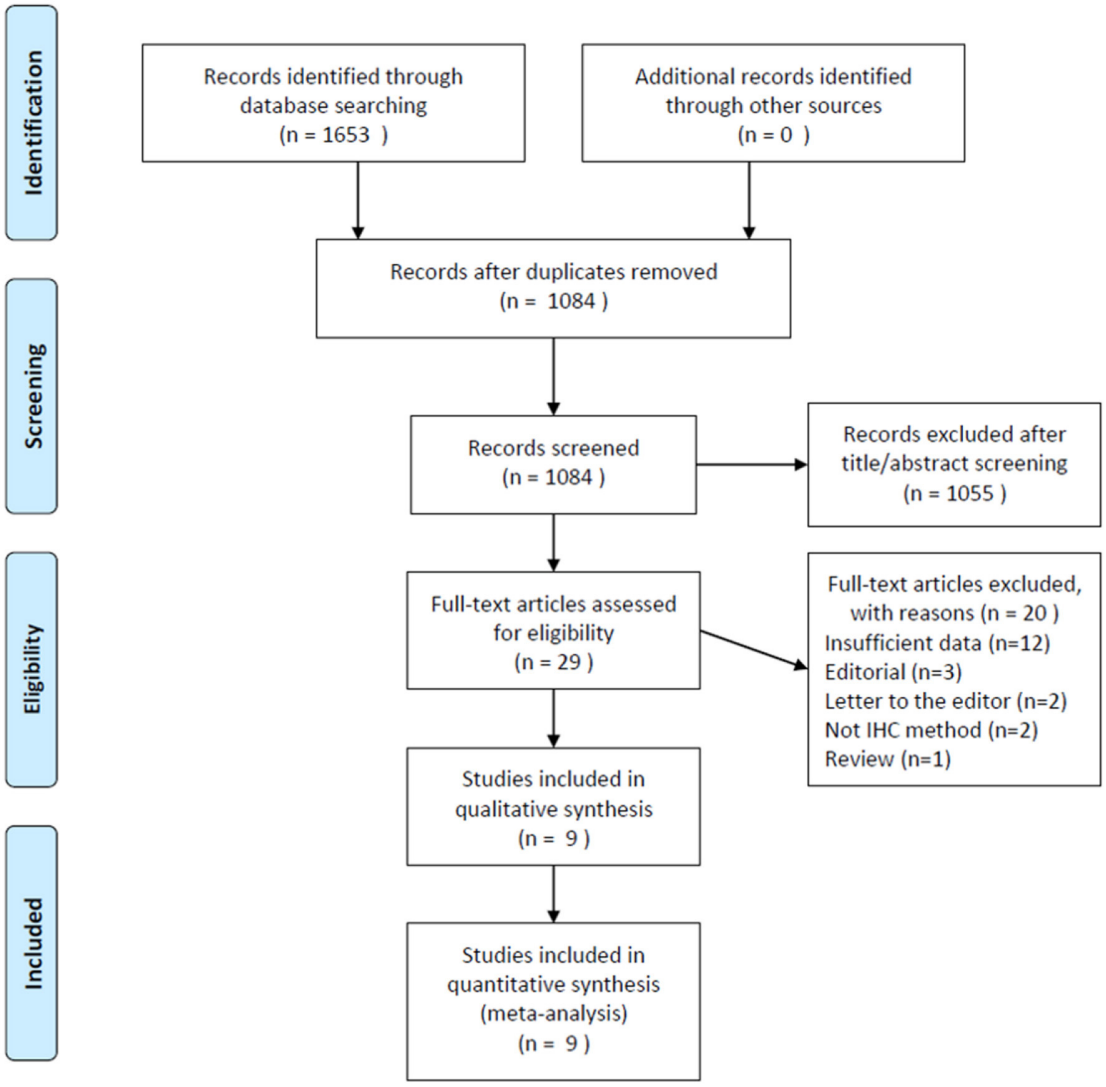
The literature search process.

The initial literature search identified 1,653 records, and after duplicate studies were removed, 1,084 articles were screened by title and abstract. Subsequently, 1,055 articles were excluded, and 29 articles were assessed by full-text examination. A total of 20 full-text articles were excluded for the following reasons: 12 lacked necessary information, three were editorials, two were letters, two did not report using an IHC method, and one was a review. Finally, nine articles (8–16) published between 2015 and 2019 were included in this meta-analysis. The selection flowchart is displayed in Figure 1.

### Study Characteristics

The baseline characteristics of the included studies are shown in Table 1. Five studies were performed in China (11, 13–16), two in Japan (8, 9), and one each in Taiwan (10), and Italy (12), respectively. The total sample size was 921, ranging from 40 to 205. All studies employed a retrospective design and used an IHC detection method. Six studies (8–11, 13, 14) used 5% as the cut-off value, and three studies (12, 15, 16) applied 1%. IHC techniques used in the included studies are shown in Table 2. All nine studies demonstrated an association between PD-L1 and OS. All studies had NOS scores of six or higher.

**Table 1:** Basic characteristics of included studies.

**Author**	**Year**	**Country/region**	**Sample size**	**Age (*y*)**	**Detection method**	**Study period**	**Stage**	**Cut-off value**	**Treatment status**	**Treatment**	**Line of chemotherapy**	**Treatment of ICIs**	**Specimen**	**Study design**	**NOS score**	**Survival outcome**
Bonanno	2018	Italy	104	68.9 (46.9–85.8)	IHC	1996–2015	I–III: 66 IV: 38	1%	Mixed	Stage I–III: Surgery or chemoradiotherapy Stage IV: Chemotherapy	First line	No	Tumor cells	Retrospective	7	OS
Chang	2017	Taiwan	186	67.1 (36–89)	IHC	2010–2015	I–III: 74 IV: 112	5%	Treatment naive	Chemotherapy, radiotherapy, or combination chemoradiotherapy	First line	No	Tumor cells	Retrospective	8	OS
Ishii	2015	Japan	102	70 (36–85)	IHC	2002–2013	I–IV:102	5%	Treatment naive	I–III: Chemoradiotherapy, chemotherapy, and surgery+ chemotherapy IV: Platinum-based chemotherapy	First line	No	Tumor cells	Retrospective	7	OS
Jing	2018	China	61	56 (30–74)	IHC	2009–2011	I–III: 61	5%	Treatment naive	Surgery	No	No	Tumor cells	Retrospective	8	OS
Liu	2018	China	80	54 (34–72)	IHC	2010–2012	I–III: 80	5%	Treatment naive	Surgery	No	No	Tumor cells	Retrospective	7	OS
Miao	2017	China	83	59 (35–84)	IHC	2010–2012	I–III: 47 IV: 36	5%	Treatment naive	I–III: Chemoradiotherapy, surgery + chemotherapy, radiotherapy, or chemotherapy IV: Chemotherapy	First line	No	Tumor cells	Retrospective	7	OS
Toyokawa	2016	Japan	40	69 (48–84)	IHC	1974–2015	I–III: 40	5%	Treatment naive	Surgery	No	No	Tumor cells	Retrospective	7	OS
Xu	2019	China	60	NA	IHC	2008–2014	I–III: 60	1%	Treatment naive	Surgery	No	No	Tumor cells	Retrospective	6	OS
Zhao	2019	China	205	NA	IHC	2005–2015	I–III: 182 IV: 9	1%	Treatment naive	Surgery, neoadjuvant chemotherapy + surgery	No	No	Tumor cells	Retrospective	6	OS

*NA, not available; IHC, immunohistochemical analysis; NOS, Newcastle-Ottawa Scale; OS, overall survival; ICIs: immune checkpoint inhibitors*.

**Table 2:** Methods for PD-L1 detection in this meta-analysis.

**Author**	**Year**	**Method**	**Primary antibody**						**Cut-off value expression**
			**Antibody type**	**Antibody**	**Antibody clone**	**Antibody source**	**Antibody dilution**	**Antibody company**	
Bonanno	2018	IHC	MAB	Anti-PD-L1	22C3	NA	NA	Dako, Carpenteria, CA, USA	1%
Chang	2017	IHC	PAB	Anti-PD-L1	NA	Rabbit	1:250	Proteintech group Inc., Chicago, IL, USA	5%
Ishii	2015	IHC	MAB	Anti-PD-L1	NA	Rabbit	NA	Abcam, Cambridge, United Kingdom	5%
Jing	2018	IHC	MAB	Anti-PD-L1	SP142	Rabbit	NA	Spring Bioscience, CA	5%
Liu	2018	IHC	MAB	Anti-PD-L1	SP142	Rabbit	1:100	Spring Bioscience Corporation, Pleasanton, CA, USA	5%
Miao	2017	IHC	NA	Anti-PD-L1	SP66	NA	NA	Springbio, USA	5%
Toyokawa	2016	IHC	MAB	Anti-PD-L1	SP142	Rabbit	1:100	Spring Bioscience, Ventana, Tucson, AZ, USA	5%
Xu	2019	IHC	MAB	Anti-PD-L1	2B11D11	Mouse	1:200	ProteinTech Group, Inc., Chicago, IL, USA	1%
Zhao	2019	IHC	MAB	Anti-PD-L1	22C3	Mouse	NA	Dako, Carpenteria, CA, USA	1%

*IHC, immunohistochemical analysis; MAB, monoclonal antibody; PAB, polyclonal antibody; NA, not available*.

### Effect of PD-L1 Expression on OS

**Figure 2:**
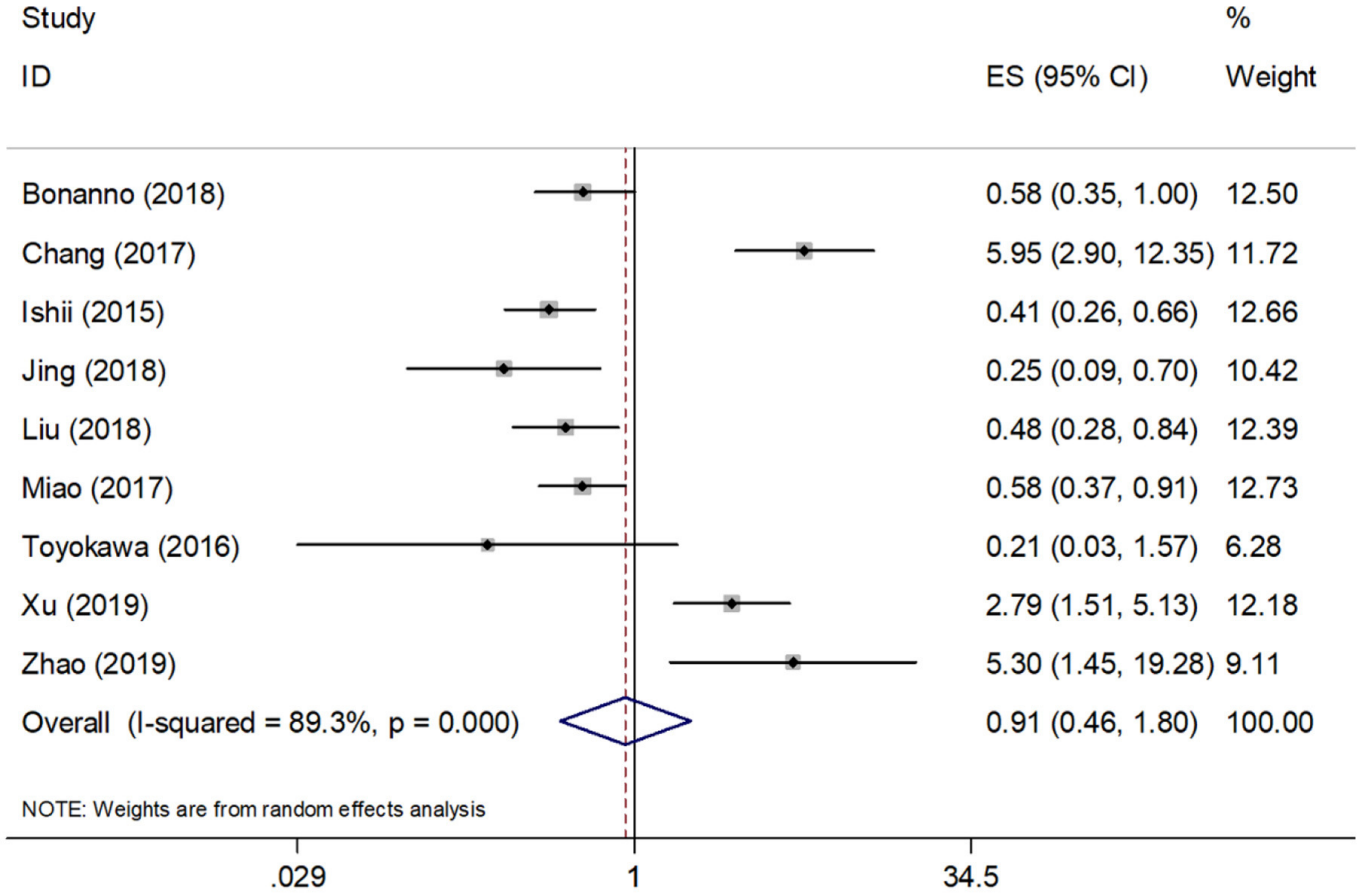
Meta-analysis of the association between PD-L1 expression and OS.

All nine studies of 921 patients demonstrated a correlation between PD-L1 and OS. Significant heterogeneity (*I*^2^ = 89.3%, *P* < 0.001) was noted; thus, a random-effects model was applied. As shown in Figure 2 and Table 3, the pooled data with HR = 0.91, 95% CI = 0.46–1.80, and *p* = 0.787 indicated that PD-L1 expression was not a significant predictor of poor OS. For further investigation, subgroup analyses divided by sample size, clinical stage, and cut-off value were also carried out. As shown in Table 3, significant heterogeneity continued to exist in all subgroups, and PD-L1 expression was not significantly associated with OS, irrespective of sample size (*n* < 100: HR = 0.61, 95% CI = 0.26–1.42, *p* = 0.514; *n* ≥ 100: HR = 1.54, 95% CI = 0.42–5.66, *p* = 0.255), clinical stage (I–III: HR = 0.59, 95% CI = 0.17–2.04, *p* = 0.402; I–IV: HR = 1.22, 95% CI = 0.48–3.10, *p* = 0.681), or cut-off value (1%: HR = 1.89, 95% CI = 0.51–7.03, *p* = 0.342; 5%: HR = 0.63, 95% CI = 0.28–1.45, *p* = 0.281).

**Table 3:** Subgroup analysis of the association between PD-L1 expression and OS in SCLC.

**Variables**	**No. of studies**	**No. of patients**	**Analysis model**	**HR (95%CI)**	***p***	***I*^**2**^ (%)**	***P*-value for heterogeneity**
OS	9	921	Random	0.91 (0.46–1.80)	0.787	89.3	<0.001
**Sample Size**							
<100	5	324	Random	0.61 (0.26–1.42)	0.514	85.3	<0.001
≥100	4	597	Random	1.54 (0.42–5.66)	0.255	93.6	<0.001
**Clinical Stage**							
I–III	4	241	Random	0.59 (0.17–2.04)	0.402	88.4	<0.001
I–IV	5	680	Random	1.22 (0.48–3.10)	0.681	91.8	<0.001
**Cut-off Value**							
1%	3	369	Random	1.89 (0.51–7.03)	0.342	89.8	<0.001
5%	6	552	Random	0.63 (0.28–1.45)	0.281	89	<0.001

### Association Between PD-L1 Expression and Clinicopathological Features

In a total of seven studies including 612 subjects, the correlations among PD-L1 expression, and six clinicopathological parameters were investigated. The clinicopathological factors were gender (male vs. female), age (≥60 vs. <60 years), pathological *N* stage (N1–2 vs. N0), pathological *T* stage (≥T2 vs. T1), serum lactate dehydrogenase (LDH) level (abnormal vs. normal), and performance status (2–3 vs. 0–1). The results are shown in Table 4. The pooled ORs and 95% CIs demonstrated that PD-L1 expression was not significantly associated with gender (OR = 1.12, 95% CI = 0.73–1.74, *p* = 0.601), age (OR = 1.15, 95% CI = 0.58–2.30, *p* = 0.683), pN stage (OR = 0.65, 95% CI = 0.24–1.72, *p* = 0.381), pT stage (OR = 1.16, 95% CI = 0.26–5.23, *p* = 0.847), serum LDH level (OR = 1.06, 95% CI = 0.13–8.43, *p* = 0.958), or performance status (OR = 0.69, 95% CI = 0.24–1.95, *p* = 0.479).

**Table 4:** Meta-analysis of the association between PD-L1 expression and clinicopathological features of SCLC.

**Characteristics**	**No. of studies**	**No. of patients**	**Analysis model**	**OR (95%CI)**	***p***	***I*^**2**^ (%)**	***P*-value for heterogeneity**
Gender (male vs. female)	7	612	Fixed	1.12 (0.73–1.74)	0.601	0	0.503
Age (≥60 vs. <60)	7	612	Random	1.15 (0.58–2.30)	0.683	65.5	0.008
p N stage (N1-2 vs. N0)	4	241	Random	0.65 (0.24–1.72)	0.381	60.2	0.057
p T stage (≥T2 vs. T1)	3	161	Random	1.16 (0.26–5.23)	0.847	78.5	0.01
Serum LDH level (abnormal vs. normal)	3	371	Random	1.06 (0.13–8.43)	0.958	87.5	<0.001
Performance status (2–3 vs. 0–1)	2	185	Fixed	0.69 (0.24–1.95)	0.479	0	0.706

*LDH, lactate dehydrogenase*.

### Sensitivity Analysis

**Figure 3:**
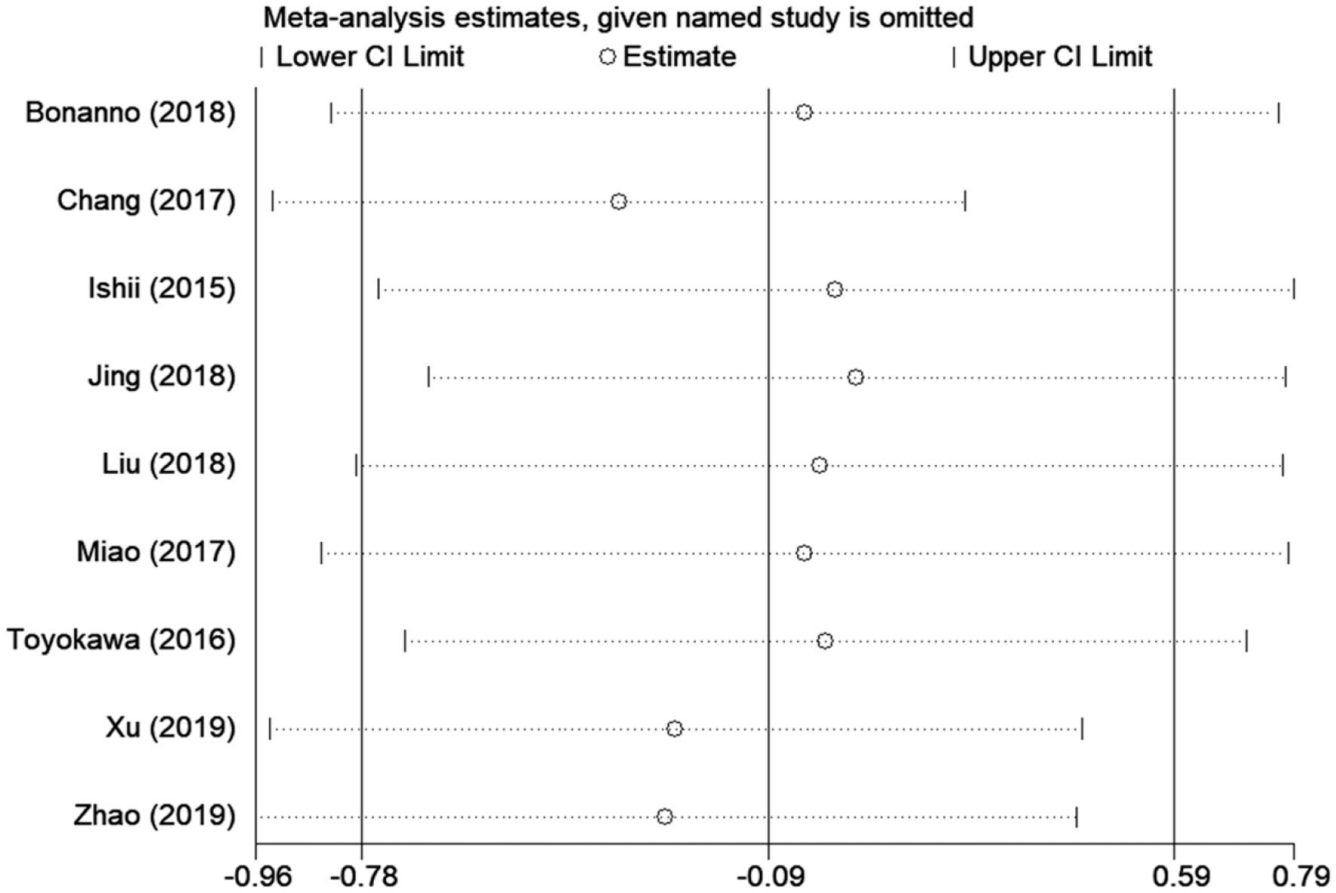
Sensitivity analysis of PD-L1 and OS.

Sensitivity analysis was carried out by deletion of each study in turn to evaluate their influences on the overall HR. Omission of each study did not substantially alter the pooled HR, indicating the stability and credibility of the results (Figure 3).

### Publication Bias

**Figure 4:**
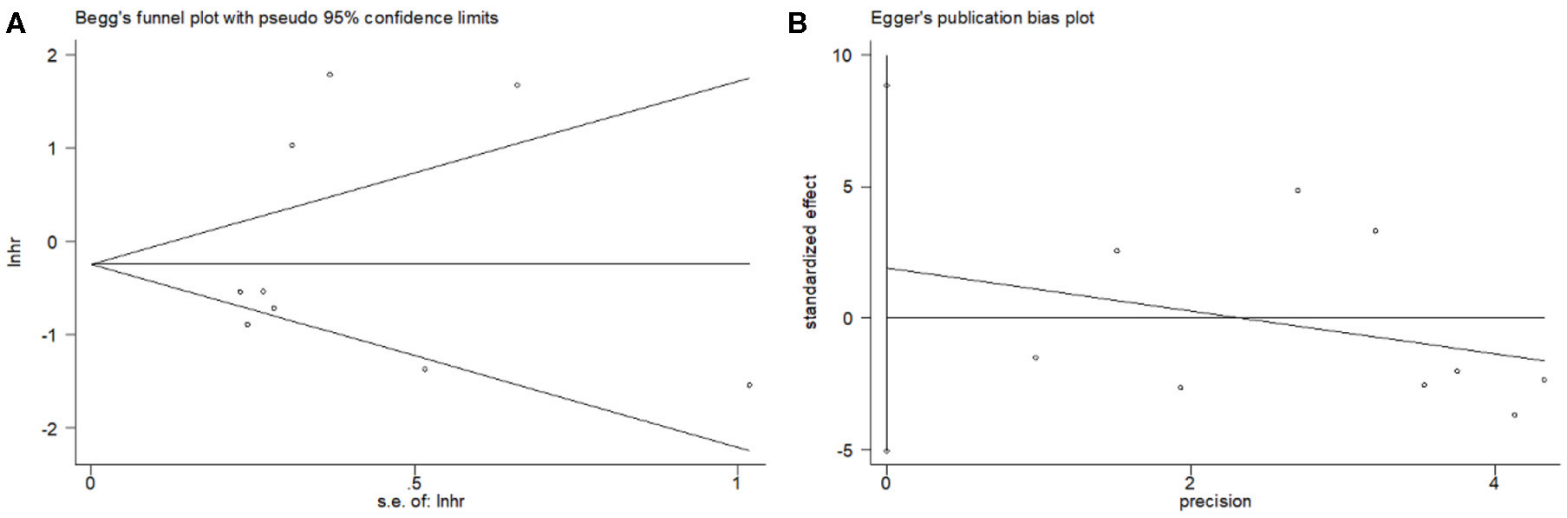
Publication bias tested by **(A)** Begg's test and **(B)** Egger's test for OS.

Both Begg's and Egger's tests were applied to detect potential publication bias in this study. As shown in Figure 4, the Begg's funnel plots appeared symmetrical (Begg's *p* = 0.466), and the *p*-value in Egger's test was 0.539. The results showed no significant publication bias in this meta-analysis.

## Discussion

SCLC is a malignant cancer due to its fast growth, tendency to metastasize, and chemotherapy resistance (22). In addition, ~65% of new patients are at an extensive stage when diagnosed, and <5% of those patients survive for 2 years (23, 24). Several ongoing phase I, II, and III clinical trials have revealed the primary results of PD-1 inhibitors for SCLC (25, 26). In the present meta-analysis, nine relevant studies were included to explore the prognostic impact of PD-L1 expression on SCLC. According to the pooled results, we failed to find an independent prognostic role of PD-L1 in OS. Moreover, PD-L1 expression showed no significant association with any clinicopathological features of SCLC. Taken together, these results suggest that PD-L1 may not play a significant role in the prognostication of SCLC. To our knowledge, this study is the first meta-analysis on the prognostic effect of PD-L1 in SCLC.

The PD-1/PD-L1 checkpoint inhibitors, as an important part of immunotherapy, show promising effects in patients with SCLC as the first-line treatment (27). The IMpower133 trial showed that first-line atezolizumab plus chemotherapy prolongs the median OS (12.3 vs. 10.3 months, *p* = 0.007) and median progression-free survival (5.2 vs. 4.3 months, *p* = 0.02) compared with those following standard chemotherapy alone in patients with extensive-stage SCLC (7). Another phase II trial (IFCT-1603 trial) revealed that atezolizumab monotherapy in patients with relapsed SCLC fails to show significant efficacy compared to that of chemotherapy (*p* = 0.6) (25). However, the safety of atezolizumab is acceptable, as no unexpected safety concerns are observed (25). Several ongoing trials may provide further evidence of the efficacy of PD-L1 checkpoint inhibitors for SCLC in the near future (28–30).

In the present meta-analysis, the data demonstrated that PD-L1 overexpression is not associated with poor prognosis in patients with SCLC. Our findings are in line with a recent study of the prognostic role of PD-L1 in patients with oral squamous cell carcinoma (31). In Troiano's study, 10 studies involving a total of 1,060 patients were included in their meta-analysis (31). The pooled results suggested that PD-L1 expression is not associated with poor OS (*p* = 0.10), disease-free survival (*p* = 0.40), disease-specific survival (*p* = 0.29), or lymph node metastasis (*p* = 0.53). These results were similar to our results. However, the results of each included study were different. In Troiano's study, almost all the included articles reported non-significant results with the 95% CIs overlapping in the OS analysis (31). Meanwhile, in our meta-analysis, the HRs of most studies were significant. However, eligible studies presented a contrary tendency in the HRs, resulting in the negative results of the total analysis. These findings suggest that PD-L1 expression may show very distinct prognostic function in patients with SCLC. Many previous meta-analyses have suggested a significant association between PD-L1 and poor prognosis in various tumors, including those of cervical cancer (32), renal cell carcinoma (33), pancreatic cancer (34), and breast cancer (35). It has been shown that journals seem to prefer reporting significant results (36), which may introduce publication bias. Notably, in the current meta-analysis, all included studies did not enroll patients receiving ICIs; therefore, whether PD-L1 expression is associated with the efficacy of ICIs and OS in patients with SCLC remains unclear. In a recent meta-analysis (37, 38), evidence showed that PD-1/PD-L1 inhibitors significantly improve OS in patients with either PD-L1 positivity or PD-L1 negativity compared with controls (37, 38). However, in these meta-analyses (37, 38), patients with SCLC were not included; therefore, further studies should be conducted to investigate the impact of PD-L1 expression on the prognosis of patients with SCLC receiving ICIs.

Although we performed this meta-analysis based on the PRISMA guidelines, several limitations must be acknowledged. First, significant heterogeneity was observed in the OS analysis. Although subgroup analysis was conducted, there remained significant heterogeneity. Second, the antibodies of PD-L1 and cut-off values were not uniform in the included studies, which may have caused inherent heterogeneity in the meta-analysis. All the included studies used 1 or 5% as the cut-off value, whereas the cut-off value of PD-L1 expression was not uniform in various studies. In the KEYNOTE-024 study (39), which used PD-L1 expression >50% as the cut-off value, the data showed that first-line pembrolizumab monotherapy continues to demonstrate an OS benefit over chemotherapy in patients with previously untreated advanced non-small cell lung cancer without epidermal growth factor receptor/anaplastic lymphoma kinase aberrations. Although the KEYNOTE-024 study (39) was not included in our meta-analysis, we also suggest a uniform cut-off value of PD-L1 expression in patients with SCLC to render the results of different studies more comparable. Third, all the included studies were of a retrospective study design. Although we did not limit the study design (prospective or retrospective) in the inclusion and exclusion criteria, and we also checked relevant prospective trials (40, 41) and meta-analyses (37, 38), we failed to identify prospective studies in our meta-analysis because of insufficient data in those studies. We suggest that the prognostic value of PD-L1 expression in SCLC should be explored in prospective trials. Fourth, all the included studies explored the prognostic value of PD-L1 in the whole group of included patients with SCLC, and more detailed investigations were not performed. For example, in the nine included studies in this meta-analysis, four studies enrolled patients with stage I-III (limited stage) (9, 13–15) and five studies recruited patients with stage I-IV (both limited stage and extensive stage) (8, 10–12, 16). We conducted subgroup analysis on patients with stage I-III by pooling data of four studies and on stage I-IV from five studies. In clinical practice, the prognostic value of PD-L1 for patients with stage IV (extensive stage) was very important, however, the needed data are not available in the five studies on stage I–IV (8, 10–12, 16). Those five studies only provide the data of association of PD-L1 and survival in whole patients group (stage I–IV as a whole). The data of PD-L1 and prognosis of patients with stage IV was not provided. The data of PD-L1 expression in different subgroups [i.e., stage IV vs. stage I–III; patients with stage I–III cancer treated with chemoradiation with or without prophylactic cranial irradiation (PCI); patients with stage I cancer treated with surgery and adjuvant chemotherapy with or without PCI] were not available in the included articles. Therefore, we suggest that these issues be investigated in further retrospective or prospective studies.

## Conclusions

In conclusion, this meta-analysis suggests that PD-L1 expression is not a significant prognostic factor of poor survival in SCLC. In addition, PD-L1 expression also shows no significant association with any clinicopathological features in SCLC. There were significant variations in the included studies; therefore, large-scale randomized trials are needed to validate our results.

## Data Availability Statement

All datasets generated for this study are included in the article/supplementary material.

## Author Contributions

HC and HZ collected and analyzed the data and wrote the paper. HZ collected and analyzed the data. YJ revised the whole paper. All authors reviewed the final paper, read and approved the final manuscript.

## Conflict of Interest

The authors declare that the research was conducted in the absence of any commercial or financial relationships that could be construed as a potential conflict of interest.
